# Can the state of cancer chemotherapy resistance be reverted by epigenetic therapy?

**DOI:** 10.1186/1476-4598-5-27

**Published:** 2006-07-10

**Authors:** Carlos Perez-Plasencia, Alfonso Duenas-Gonzalez

**Affiliations:** 1 Unidad de Investigación Biomédica en Cáncer, Instituto de Investigaciones Biomédicas (IIB)/Instituto Nacional de Cancerología, Universidad Nacional Autónoma de Mexico, Mexico City, Mexico

## Abstract

**Background:**

Transcriptome analysis shows that the chemotherapy innate resistance state of tumors is characterized by: poorly dividing tumor cells; an increased DNA repair; an increased drug efflux potential by ABC-transporters; and a dysfunctional ECM. Because chemotherapy resistance involves multiple genes, epigenetic-mediated changes could be the main force responsible of this phenotype. Our hypothesis deals with the potential role of epigenetic therapy for affecting the chemotherapy resistant phenotype of malignant tumors.

**Presentation of the hypothesis:**

Recent studies reveal the involvement of DNA methylation and histone modifications in the reprogramming of the genome of mammalian cells in cancer. In this sense, it can be hypothesized that epigenetic reprogramming can participate in the establishment of an epigenetic mark associated with the chemotherapy resistant phenotype. If this were correct, then it could be expected that agents targeting DNA methylation and histone deacetylation would by reverting the epigenetic mark induce a global expression profile that mirror the observed in untreated resistant cells.

**Testing the hypothesis:**

It is proposed to perform a detailed analysis using all the available databases where the gene expression of primary tumors was analyzed and data correlated with the therapeutic outcome to determine whether a transcriptome profiling of "resistance" is observed. Assuming an epigenetic programming determines at some level the intrinsic resistant phenotype, then a similar pattern of gene expression dictated by an epigenetic mark should also be found in cell acquiring drug resistance. If these expectations are meet, then it should be further investigated at the genomic level whether these phenotypes are associated to certain patterns of DNA methylation and chromatin modification. Once confirmed the existence of an epigenetic mark associated to either the intrinsic or acquired chemotherapy resistant phenotype, then a causal association should be investigated. These preclinical findings should also be tested in a clinical setting.

**Implications of the hypothesis:**

Our hypothesis on the ability of epigenetic therapy to revert the epigenetic changes leading to a transcritome profile that defines the resistant state will eventually be a more rational and effective way to treat malignant tumors.

## Background

In the cancer arena, the phenomenon of chemotherapy resistance has being for years, the focus of intensive research with the aim to increase its understanding and hopefully for finding out novel therapies to overcome it. In the past years, and before the introduction of methods for global genomic analysis, the phenomenon was mostly studied at the single gene level, for instances, the mdr gene and related members [1]; the detoxifying systems such as the glutathione-S-transferase families [2]; and antiapoptotic genes [3] just to mention some. Nevertheless, these models turned out to be a quite simplistic approximation of the chemotherapy resistance as shown by in general poor clinical results of agents against individual targets such as mdr blockers [4,5].

Thus, the chemotherapy resistant phenotype of cancer cells is by no means the result of a single gene alteration; hence, the phenomenon should be studied at a global genomic level. Such an assumption is backed by the knowledge that cancer cells do contain multiple gene genetic defects, namely mutations, deletions, duplications, translocations, etc, as well as epigenetic aberrations, such as alterations at DNA methylation and histone post-transductional modifications. Both the genetic and epigenetic alterations are not static and must act in concert in order to maintain the malignant "homeostasis" and also to cope with the cytotoxity induced by chemotherapy or radiation therapy [6,7].

Cancer is a highly heterogeneous disease however; most malignant tumors share the ability of being intrinsically resistant to chemotherapy and to acquire it during treatment. Microarray technology has the potential to better assess the complexity, redundancy and interdependence in biological pathways involved in drug resistance however, most high-throughput gene expression profiling studies with microarrays are being directed to dissect the molecular cancer pathways in the aim to improve current tumor classifications and to discover novel diagnostic and therapeutic potential targets [8-10]. Common to all of these studies is the fact that models investigated do not address mechanisms that contribute to innate drug resistance, but rather test hypotheses on how drug exposure induce resistance states. A recent study on transcriptional profiles of clinical samples collected from colorectal cancer patients prior to their exposure to a combined chemotherapy shows that 679 genes discriminates between drug resistance and sensitivity states. In addition, through transcriptome analysis and functional annotation, authors were able to suggest that the innate resistant state may be characterized by: poorly dividing tumor cells as deduced from observed accumulation of cells in mid-G1 phase and decreased DNA replication processivity; an increased DNA repair associated with cell cycle delay in late S and G2 phases preventing occurrence of mitotic catastrophe and cell apoptosis; an increased drug efflux potential by ABC-transporters pre-existing to drug exposure; and a dysfunctional extracellular matrix (ECM) with decreased renewal ability of ECM and basement membrane components, most likely resulting in decreased stimulation of angiogenesis [11].

These data clearly demonstrate that chemotherapy resistance requires of changes in expression of a large number of genes. In this regard, clonal selection of cells by genetic changes would hardly explain the resistant phenotype, instead epigenetic-mediated changes could be the driving force as by one hand, they provide a mechanism whereby expression of multiple genes could be affected simultaneously and secondly, epigenetic changes can be a rapid process as a result, resistance may therefore arise rapidly following treatment with chemotherapy in the case of acquired resistance [12-14]. In contraposition of genetic defects, the reversible nature of the epigenetic aberrations constitutes a very attractive therapeutic target and as such, a number of inhibitors of DNA methylation and histone deacetylases are currently being evaluated in cancer therapy either alone or in combination, as there is clear that these drug have a synergistic effect upon gene expression and tumor growth [15-17]. A recent study [18] reported on the global gene expression profile of the resistant colon carcinoma cell line SW480 [19-21] induced by the combined treatment of hydralazine, a DNA methylation inhibitor with valproic acid, a histone deacetylase inhibitor. The gene expression profile from the resistant colon carcinoma tumors and from the resistant colon cancer cell line treated with epigenetic agents led us to put forward the hypothesis on the potential role that epigenetic therapy has to revert the chemotherapy resistant phenotype of malignant tumors.

## Presentation of the hypothesis

Epigenetic control of gene activity is widespread in the genome of eukaryotic cells and leads to persistent gene silencing or gene expression. This control is implemented by changes in the methylation status of DNA and by chromatin modifications. The epigenetic modifications efficiently control the state of gene expression in the genome by inducing stable silencing of some genes and promoting activation of others [22,23]. The epigenetic control of gene expression is heritable through cell division, but reversible, because it does not involve DNA sequences. Recent studies reveal the involvement of DNA methylation and histone modifications in the reprogramming of the genome of mammalian cells in cancer [24-26]. In this sense, it can be hypothesized that epigenetic reprogramming can participate in the establishment of an epigenetic mark associated with the chemotherapy resistant phenotype. If this were correct, then it could be expected that agents targeting DNA methylation and histone deacetylation would by reverting the epigenetic mark induce a transcriptome profile that mirror the observed in untreated resistant cells.

**Figure 1:**
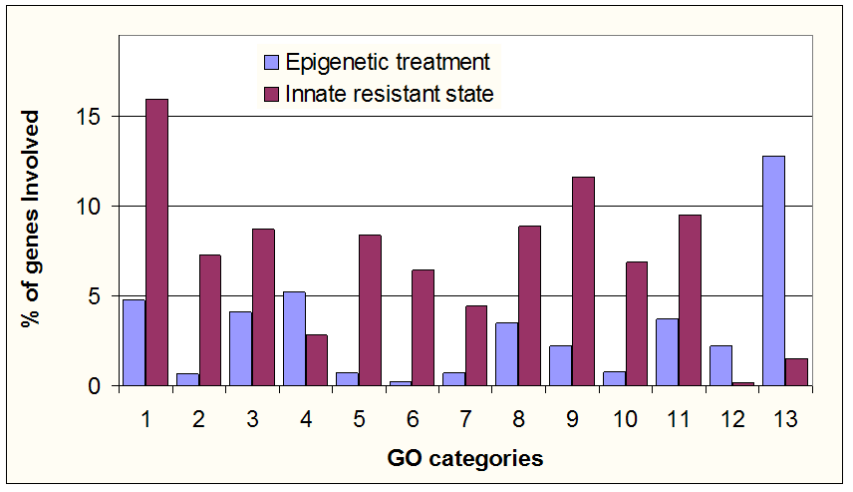
Main Gene Ontology terms which are inversely regulated in both phenotypes. We used information from "additional files" listing gene expression profile from Gaurdens et al, [11] and Chávez-Blanco et al, [18] then we took 317 and 352 up-regulated genes respectively and by means of FatiGO, comparisons in functional annotations were made. 1. Cell cycle; 2. Response to DNA damage; 3. Cell proliferation; 4. Cell adhesion; 5. M phase; 6. DNA repair; 7. Regulation of cell proliferation; 8. Regulation of cell cycle; 9. DNA metabolism; 10. M phase of mitotic cell cycle; 11. Regulation of progression through cell cycle; 12. Immune cell activation; 13. Ubiquitin Cycle.

This hypothesis surges from the comparisons in the gene expression profiling between tumors intrinsically resistant to chemotherapy [11] and the expression data from the colon carcinoma cell line SW480 treated with the DNA methylation and the histone deacetylase inhibitor hydralazine and valproic acid respectively [18] employing FatiGO module from Babelomics suite [27,28]. FatiGO is a web based tool which map biological knowledge on sets of genes to extract relevant gene ontology (GO) terms for a group of genes with respect to a reference set. The terms are considered to be relevant upon the application of a Fisher's exact test or 2 × 2 contingency tables. We observed that several cellular processes were inversely regulated as shown in Figure 1 where it seems to be a mirror image between these data. To make this comparison, we took only up-regulated genes reported as supplementary information by Gaurdens et al, (317 genes) and by Chavez-Blanco et al, (352 genes). Both lists of genes were downloaded from these publications [11,18] and then data loaded into the FatiGO data mining tool. The retrieved information was then submitted for finding differences in the distribution of GO biological process terms between both groups of genes. The biological processes selected and showed in Figure 1 were taken as significant if *p *values < 0.01; except for cell proliferation and cell adhesion terms which showed a *p *value of 0.15 and 0.23, respectively.

## Testing the hypothesis

The analysis of the published data concerning the expression patterns in colorectal carcinoma patients and the colon carcinoma cell line SW480 strongly suggests that the potential ability of epigenetic agents to revert and/or at least to interfere with the epigenetic functioning characterizing the resistant phenotype should be further studied. In this aim, it is proposed to perform a detailed analysis using available databases where the gene expression of primary tumors was analyzed and data correlated with the therapeutic outcome. It means data that from these studies aimed to the pre-treatment classification into resistant and sensitive tumor should be dissected and compared to determine whether the observation raised is a generalizable phenomenon.

It has been clearly shown that epigenetic changes occur rapidly upon chemotherapy exposure, thus it can be anticipated that assuming an epigenetic programming determines at some level the intrinsic resistant phenotype, then a similar pattern of gene expression dictated by an epigenetic mark should also be found in cell acquiring drug resistance. In fact, there are many evidences on the rapid changes in DNA methylation and chromatin modifications after cytotoxic chemotherapy in several models [29-34]. If these expectations are meet when tested with the appropriate tools, then it should be further investigated at the genomic level whether these phenotypes are associated to certain patterns of DNA methylation and chromatin modification. In this regard, there are now several powerful platforms for this kind of analysis.

Once confirmed the existence of an epigenetic mark associated with either the intrinsic or acquired chemotherapy resistant phenotype, then a causal association should be investigated. In this regard, chemotherapy resistant malignant cells should be treated with epigenetic agents to then analyze changes at the phenotype (restoration of sensitivity), transcriptome and epigenetic marks at global level. So far, a number of preclinical studies have demonstrated that either DNA methylation [35,36] of histone deacetylase inhibitors [37,38] or in combination [18], reverse drug resistance or increase the cytotoxicity of anticancer drugs and radiation. In this regard, currently there are a number of clinical trials testing epigenetic drugs either alone or in combination with conventional cytotoxic and radiation therapy [39]. Multisampling of primary tumors and/or peripheral blood is fundamental for evaluating the reversion of the epigenetic changes and their correlation with patient tumor response and prognosis. Along with all these investigations proposed, a better dissection of the contribution of specific epigenetic alterations will require an individual analysis of each known epigenetic player such as global DNA methylation, promoter DNA methylation, specific histone modification (acetylation, methylation, phosphorylation, etc) as well as changes at other molecules of the epigenetic machinery such as polycomb and trithorax groups [40].

## Implications of the hypothesis

The confirmation of this hypothesis has obvious implications in the field of cancer therapy. Generally speaking, we can name the current era of targeted therapies as ones directed against a single gene or single gene product, or in other words, "*a single gene approach*". This approach as such, has limitations because of the plasticity of tumor cell genomes as well as because of the multistep nature of cancer development where no single genetic mutation is responsible for the malignant phenotype (except for some specific neoplasms). This means that even though the blocking of a single or few abnormalities can induce to the tumor cell to stop or slow its proliferation, to induce its differentiation or to undergo apoptosis, eventually tumor cells will develop resistance to the therapy because of its ability to bypass such blocking effect by making the appropriate epigenetic changes modifying the transcriptome. In this sense our hypothesis dealing with the ability of blocking or reverting the machinery responsible of these cell strategies to survive will eventually be a more rational and effective way to deal with the resistant state of malignant tumors.

## Conclusion

The chemotherapy resistant state of cancer cells seems to be a polygenic phenomenon where epigenetic-mediated changes could be the driving force leading to this phenotype. Our hypothesis deals with the potential role of epigenetic therapy for affecting the chemotherapy resistant phenotype of malignant tumors. Preclinical information from experimental models using DNA methylation and histone deacetylase inhibitors suggests that epigenetic therapy could erase the epigenetic mark associated with the chemotherapy resistant phenotype and therefore sensitize tumors to chemotherapy.

## Competing interests

The author(s) declare that they have no competing interests.

## Authors' contributions

C P-P performed the data analysis, both authors conceived and discussed the data, A D-G wrote the manuscript.
